# Psychopathology Assessment Methods Revisited: On Translational Cross-Validation of Clinical Self-Evaluation Scale and fMRI

**DOI:** 10.3389/fpsyt.2018.00021

**Published:** 2018-02-08

**Authors:** Drozdstoy Stoyanov, Sevdalina Kandilarova, Stefan Borgwardt, Rolf-Dieter Stieglitz, Kenneth Hugdahl, Stefan Kostianev

**Affiliations:** ^1^Research Complex for Translational Neuroscience, Medical University Plovdiv (MUP), Plovdiv, Bulgaria; ^2^Department for Psychiatry and Medical Psychology, Medical University Plovdiv (MUP), Plovdiv, Bulgaria; ^3^University of Basel, Basel, Switzerland; ^4^Department of Biological and Medical Psychology, University of Bergen, Bergen, Norway; ^5^Division of Psychiatry, Haukeland University Hospital, Bergen, Norway; ^6^Department of Radiology, Haukeland University Hospital, Bergen, Norway; ^7^Department of Pathophysiology, Medical University Plovdiv (MUP), Plovdiv, Bulgaria

**Keywords:** psychopathology, functional neuroimaging, translational medical research, neuroscience, depression

## Abstract

We present in this article a study design that combines clinical self-assessment scale, simultaneously administered with fMRI data acquisition. We have used a standard block-design with two different conditions. Each active block consisted of four text statements (items), alternating diagnostically specific (DS) blocks comprising items from von Zerssen depression scale and diagnostically neutral (DN) blocks with items from a questionnaire about general interests. All items were rated on four degree Likert scale, and patients provided responses with corresponding four buttons during the fMRI session. Our results demonstrated that in healthy controls, contrasting the two types of stimuli yielded no residual activations, e.g., the DS did not produce significantly different activations compared to the DN stimuli. Furthermore, the correlation analyses did not find a relationship between brain activations and the total score of the DS statements in this group. However, contrasting the DS stimuli to the DN stimuli in the patients produced significant residual activations in several brain regions: right pre- and postcentral gyrus (including right supramarginal gyrus), left middle frontal gyrus, triangular part of the left inferior frontal gyrus and middle temporal gyrus. The left precuneus demonstrated correlations with the patients’ DS score. In the between-group comparisons, we found residual activations in the right pre- and postcentral gyrus, right supplementary motor area, medial segment of the right precentral gyrus, right superior parietal lobule, left middle frontal gyrus, left superior frontal gyrus, left occipital pole. Our results confirm the possibility of translational cross-validation of a clinical psychological test (von Zerssen’s depression scale) and fMRI. At this stage, however, we can only confirm the sensitivity of the method (its ability to distinguish healthy controls from depressed patients), but we cannot conclude anything about its specificity (distinction from different psychopathology conditions).

## Introduction

Modern psychopathology has been in a long-term crisis from different perspectives (1). One critical issue which contributes to this is the problem of validity of diagnostic methods and current classifications, which is entailed to some extent from the methodological gap existing between psychopathology and neuroscience, and functional neuroimaging in particular (2–5).

The larger body of functional neuroimaging data was collected in fMRI studies using event-related or block designs where participants had to engage in different tasks. The most common task-related fMRI studies obtain sequential scans while subjects are doing/viewing either cognitive tasks (like Stroop’s test) or emotionally valenced (sad, happy, angry, fearful) and neutral pictures. Common practice in clinical fMRI research is to perform a clinical assessment before and after the scanning that is subjected to a *post hoc* correlational analysis. This creates a temporal gap between the two measurements. In some cases, this may affect the consistency of such correlations (for example in bipolar patients with rapid cycling). Thus, the fMRI imaging and the clinical assessment may reflect different emotional states in such settings. Furthermore, the stimuli that are typically presented during an fMRI session are often diagnostically irrelevant, i.e., they cannot be incorporated directly into diagnostically valid operational procedures.

To this end unfortunately fMRI-techniques have provided rather controversial data on potential markers in psychopathology. It is difficult, if not impossible to attain universal agreement on what kind of procedures should be used in order to incorporate functional neuroimaging findings into diagnostic and treatment standards in psychiatry.

Having in mind the complexity of contemporary clinical practice, we have developed a novel study design that combines clinical assessment scales, simultaneously administered with fMRI data acquisition. For a broader explanation of the underlying arguments behind the concept of translational convergent cross-validation in psychiatry, see Ref. (2, 3, 6). In the following we will shortly present the background for the design.

In our design, clinical fMRI studies involve real-time ratings of the clinical state, using disorder-relevant self-evaluation scales administered during fMRI data acquisition. Several standardized clinical self-assessment scales are used by clinicians worldwide. For example, in the case of depression, the Beck Depression Inventory (7), Zung (8), and von Zerssen (9) are some commonly used self-report scales. Those scales are validated against observer-based interviews, such as the Montgomery-Åsberg Depression Rating Scale (10), or the Hamilton Depression Scale (11), which are basically composed of similar kind of statements (items), extracted from either patient’s (first person) or professional’s (third person) narratives (6, 12). We now suggest bringing together the narrative (subjective) perspective with its objective neurobiological correlates. The suggested simultaneous fMRI data acquisition and the standardized rating scales have the potential to overcome the described temporal gap.

By implementing this new design, we expect to find significant correlations between the psychological rating scale score (total score, or score on given items or groups of items) and the pattern of blood-oxygen-level dependent (BOLD) activity. This is a critical step forward to achieve *synchronization and concordance* of the applied measures and data bases in psychiatry as defined elsewhere (3, 13, 14). Ultimately, we can re-validate the clinical assessment tools according to the evidence from the simultaneous cross-validation with the neuroimaging methods. As a consequence, we will be able to rely on inexpensive instruments for clinical assessment. We consider such a convergent design with simultaneous clinical self-evaluation and fMRI data sampling, as having potential for revealing reliable constellations of biomarkers that could ultimately inform diagnosis and choice of treatment.

We have decided to focus on depressive episode on syndromal level as more consistent and homogenous clinical construct in comparison to schizophrenia, anxiety disorders and other psychopathological phenomena. We assume that the clear nosological approach as adopted from bio-medicine would not be appropriate for validation of psychiatric classification (15). Therefore, we have recruited subjects with current depressive episode in the context of either bipolar disorder or major depressive disorder compliant with the DSM-IV TR criteria.

### Aim

The aim of the current study was thus to investigate the translational validity of von Zerssen’s depression scale and its fMRI-correlates during their simultaneous implementation in patients with depression and healthy controls.

## Materials and Methods

### Subjects

We recruited 18 adult subjects (mean age 44.3 ± 3.6 years, six males) complying with the DSM-IV-TR criteria for depressive episode (single or recurrent) in the context of major depressive disorder (12 subjects) or bipolar affective disorder (6 subjects), as assessed by the general clinical interview and the structured Mini International Neuropsychiatric Interview (M.I.N.I 6.0). Severity of current episode was assessed and only subjects with moderate to severe depression (e.g., a total score on the MADRS of at least 20 were included). Subjects were excluded if they had a second axis-I diagnosis (psychotic, anxiety, substance-related disorder), severe decompensated somatic disorder, neurological disorder, history of head trauma with loss of consciousness, severe suicidal risk (10th item of MADRS ≥ 2).

Eighteen age, sex, and education matched healthy controls (median age 39.1 ± 2.5 years, six males) were enrolled in the study as a control group. They were subjected to a general clinical interview and the structured M.I.N.I. and they were included if they did not comply with any of the DSM-IV-R diagnoses included, had no history of psychiatric disorder, neurological disorder, head trauma with loss of consciousness. All participants provided a written informed consent and the study was approved by the University’s Ethics Committee.

### MR Scanning

The scanning of the participants was done with a 3 T MRI system (GE Discovery 750w). The MR protocol included a structural scan [Sag 3D T1 FSPGR, slice thickness 1 mm, matrix 256 × 256, TR (relaxation time) −7.2 msec, TE (echo time) −2.3, flip angle 12°], and a functional scan (2D EPI, slice thickness 3 mm, matrix 64 × 64, TR −2,000 ms, TE −30, flip angle 90°). Before each functional scan, five dummy time series were acquired.

### Experimental Procedure

We used a standard block-design with two different “ON” conditions and one “OFF” condition, with a total duration of 8 min and 32 s Each “ON” block consisted of four text statements presented for 8 s each on LCD screen. Diagnostically specific (DS) blocks consisted of 4 consecutive statements from the von Zerssen depression scale (“I cry easily,” “I am more sensitive to criticism than I was before”) and the diagnostically neutral (DN) blocks consisted of four statements from a questionnaire about genral interests and likes (such as “I like to write books or plays,” “I like to repair household appliances,” etc.). Under each statement four possible item responses were presented as well as the four buttons corresponding to the responses (completely true = upper left, mostly true = lower left, somewhat true = lower right, not true = upper right button). There were four blocks of each type, alternating between DS and DN conditions, and each ON block was followed by an “OFF” block with a fixation cross in the middle of the screen (DS_OFF_DN_OFF_DS_OFF…). The duration of ON and OFF blocks was 32 s. For the active conditions, the participants were instructed to read the statements carefully and to respond with a button press according to their level of agreement, and for the passive OFF condition, to focus on the fixation cross without thinking of anything in particular.

### fMRI Data Analysis

Data were analyzed using the SPM 12 (Statistical Paramertic Mapping, http://www.fil.ion.ucl.ac.uk/spm/) software running on MATLAB R2015 for Windows. The preprocessing included the following steps: (i) realignment of the functional data for correction of head motion, (ii) coregistration between the high-resolution anatomical image and the functional scans, (iii) intra-individual estimation of spatial registration parameters based on the anatomical image and (iv) transformation of the coregistered functional data to standardized Montreal Neurological Institute (MNI) space, followed by (v) spatial smoothing with a 6 mm full-width-at-half-maximum Gaussian kernel.

The model for first-level analysis was then specified, parameters estimated and t-contrasts defined for the active vs. passive conditions, with the contrasts (DS > DN) and the (DN > DS), respectively. The resulting contrast maps from each comparison and for each subject were then used in a second-level random-effects analysis for between-group differences (patients > controls and controls > patients), and for the interaction of groups × conditions. The level of significance was set to *p* > 0.05 false discovery rate (FDR) corrected.

### Behavioral Data Analysis

Correlations were tested for the total score from the DS statements and both the DS > DN and DN > DS contrast maps. Demographic and clinical characteristics of the subjects were analyzed by means of SPSS 22.0 for Windows. The level of significance for all tests was set to *p* < 0.05. Because of the small sample size, we used the Mann–Whitney test for comparison of continuous variables, Chi-square and Fisher’s exact test for testing of categorical variables.

## Results

### Demographic and Clinical Characteristics

There were no statistically significant differences in age, sex and education between the two groups. Expectedly, patients had significantly higher MADRS and von Zerssen score (Table 1).

**Table 1:** Demographic and clinical characteristics.

	Healthy controls (*n* = 18)	Patients (*n* = 18)	*p*-Value
Age	39.1 ± 2.5	44.3 ± 3.6	0.308^a^
Sex (M:F)	6:12	6:12	1.0^b^
Education (secondary:higher)	8:10	8:10	1.0^b^
MADRS score	3 (2–3.5)	29.7 (25.7–33.7)	*0.001^a^
Von Zerssen score	6 (4–8)	25 (22–29)	*0.001^a^

*^a^Mann–Whitney U test*.

*^b^Fisher’s exact test*.

*p <0.005.

*IQR, interquartile range; MADRS, Montgomery–Åsberg Depression Rating Scale*.

### fMRI Results

The first-level of analysis provided four contrast maps (DS > DN, DN > DS, DS > OFF, DN > OFF) for each subject, which were used in the second-level within- and between-group analyses. The *t*-contrast for the within-group DS vs. DN blocks yielded no residual activations in the healthy controls, while the patients showed higher activation in several clusters located in the right pre- and postcentral gyri (MNI coordinates 32, −14, 66 and 48, −22, 58), left middle frontal gyrus (−40, 16, 30), left middle temporal gyrus (−50, −38, 0), and the left inferior frontal gyrus triangular part (−50, 22, −4).

**Figure 1:**
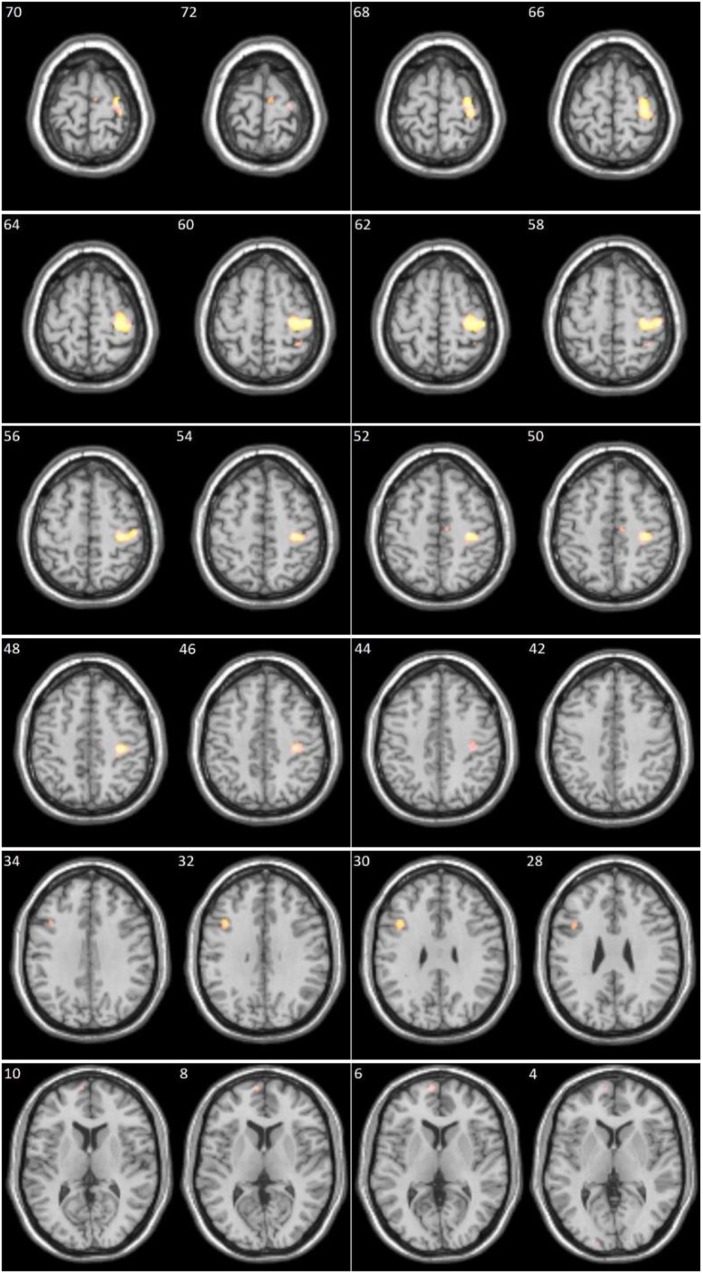
Clusters significantly more activated in patients compared to controls (numbers in left upper corner represent x axis in Montreal Neurological Institute coordinates).

For the between-group analysis we performed first a two-sample t-test on the DS > DN contrast maps. The patients demonstrated significantly higher activations in the right pre- and postcentral regions, left middle frontal gyrus, medial segment of left superior frontal gyrus, right supplementary motor area, right superior parietal lobule (See Table 2 and Figure 1 for details).

**Table 2:** Patients > Controls for the DS > DN contrast maps.

Anatomical localization	Cluster size (number of voxels)	MNI coordinates			*p*-Value (FDR-corrected)
		*x*	*y*	*z*	
Right pre- and postcentral gyrus	734	48	−22	58	0.006

		32	−24	62	

Left middle frontal gyrus	58	−40	14	30	0.009

Medial segment of left superior frontal gyrus	23	−8	64	6	0.023

Right supplementary motor area	12	8	−12	72	0.026

Left occipital pole	16	−18	−98	2	0.027

Right superior parietal lobule	14	36	−46	60	0.027

Medial segment of the right precentral gyrus	11	10	−18	50	0.030

*DS, diagnostically specific condition; DN, diagnostically neutral condition; FDR, false discovery rate; MNI, Montreal Neurological Institute*.

On the group level, the DN > DS contrast yielded no significant residual activations neither in the patients nor in the control subjects. The between-group analysis resulted, as expected, in the same significant clusters as mentioned above (right pre- and postcentral regions, left middle frontal gyrus, medial segment of left superior frontal gyrus, right supplementary motor area, right superior parietal lobule) now in control subjects > patients. While the opposite *t*-contrast, patients > controls, yielded no suprathreshold clusters.

The between-group analysis for the contrasts DS > OFF and DN > OFF, respectively, showed no statistically significant difference between the healthy controls and the patients although in each group both contrasts resulted in large clusters of activation in cortical and subcortical regions.

### Correlations between BOLD Signal and Behavioral Data

**Figure 2:**
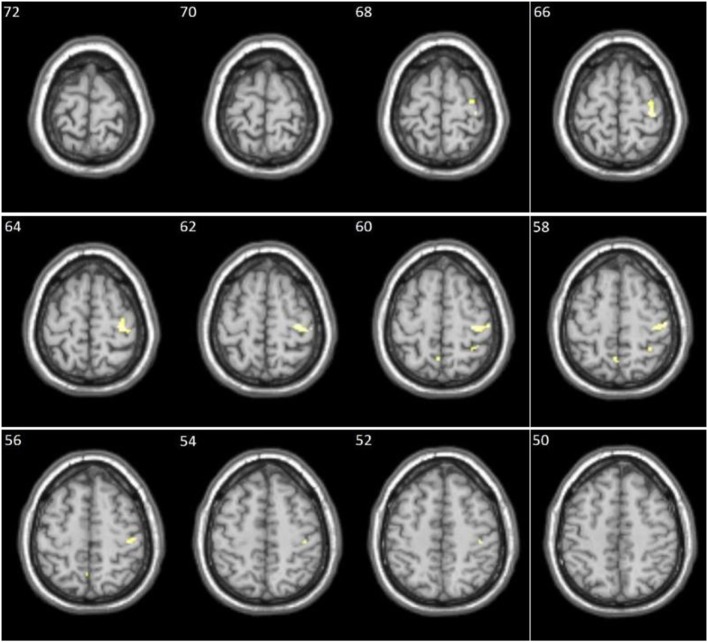
Clusters showing significant positive correlation between the diagnostically specific (DS) score and the DS > DN contrast in patients (numbers in left upper corner represent *x*-axis in Montreal Neurological Institute coordinates).

No significant correlations were found in the healthy controls between the total score of the von Zerssen scale statements and residual activations of the DS > DN contrast. For the patient group, however, there were significant positive correlations with activations in the right pre- and postcentral gyrus, as well as in left precuneus and right superior parietal lobule (Table 3; Figure 2).

**Table 3:** Positive correlations between the DS score and the DS > DN contrast in patients.

Anatomical localization	Cluster size (number of voxels)	MNI coordinates			*p*-Value (FDR-corrected)
		*x*	*y*	*z*	
Right pre- and postcentral gyrus	133	34	−14	66	0.044
		48	−22	58	

Left Precuneus	12	−2	−58	58	0.044

Right superior parietal lobule	9	34	−46	60	0.044

*DS, diagnostically specific condition; DN, diagnostically neutral condition; FDR, false discovery rate; MNI, Montreal Neurological Institute*.

## Discussion

Our results demonstrated that in healthy controls, contrasting the two types of stimuli yielded no residual activations, e.g., the diagnostically specific (DS) stimuli did not produce significantly different activations compared to the diagnostically neutral (DN) stimuli. Furthermore, the correlation analyses did not find a relationship between brain activations and the total score of the DS statements in this group.

However, contrasting the DS stimuli to the DN stimuli in the patients produced significant residual activations in several brain regions (see Table 2 and Figure 1 for details). Positive correlations were also found between the DS > DN contrast and the DS score in several activation clusters (see Table 3 and Figure 2 for details).

Changes in brain activation, metabolism of glucose, neurotransmitters such as serotonin and its receptors and even gray matter volumes in various areas of the frontal cortex have previously been reported in patients with depression (16–21), and we will discuss our findings in light of current knowledge about the functional characteristics of the brain areas in which the significant clusters were located.

Residual activations in the depressed patients in the right pre- and postcentral gyrus can be explained by the demands for motor responses during the performance of the task (pressing the buttons). Since the patients in comparison to healthy controls were responding more frequently with the left hand buttons, i.e., positive answers (completely true, mostly true, see Methods), this means that they more often used their left hand in the DS condition. We cannot exclude the hypothesis that the observed motor cortex activations might be as well interpreted in terms of changes of psychomotor behavior (agitation or inhibition) regarded as fundamental symptoms of the depressive episode. Given the sample size it is difficult to justify to what extent and in which direction does this factor influence the results.

The remaining clusters (left middle frontal gyrus, triangular part of the left inferior frontal gyrus and middle temporal gyrus) were located in areas associated with language functions, semantic processing and memory (22–24). It is reasonable to assume that the patients probably would recruit these areas to a greater extent than controls when responding to the diagnostically relevant statements.

One of the statistically significant clusters that correlated with the DS total score items in the patients overlaps with the abovementioned cluster resulting from the DS > DN contrast with peaks in the right pre- and post-central gyrus. The explanation is that the more frequent motor response with the left buttons causes the activation of this area, and at the same time this leads to a higher DS score. Further, a significant area within the same cluster was identified to be the right supramarginal gyrus (rSMG), which is previously reported to be involved in empathy (25). The correlation with activations within the right superior parietal lobule is probably also, in addition, related to regulation of working memory, and motor function, assuming that this area is implicated in visual-motor coordination (26).

The left precuneus also demonstrated correlations with the patients’ DS score, which can be explained by the variety of cognitive functions to which this area is related—visual-spatial imagery, reproduction of episodic and autobiographical memory, and self-processing operations, namely first-person perspective taking and experience of agency (27). We can assume that more frequent positive responses (higher DS scores) are accompanied by a higher degree of activation of processes related to autobiographical memory and a first-person perspective.

In the between-group comparisons, the more pronounced activations in the right pre- and postcentral gyrus are explained again by the more frequent left-handed motor response in the patients. The other clusters located in the right hemisphere—the right supplementary motor area and the medial segment of the right precentral gyrus (Brodmann area 6) and the right upper parietal lobule (Brodmann area 7) are also related to motor responses, and movement planning, and visual-motor coordination, respectively (26, 28).

The cluster with a peak in the left middle frontal gyrus (Brodmann area 8) falls into the functional area of the dorsolateral prefrontal cortex (DLPFC) associated with executive functions, such as attention, working memory, planning, and inhibition of response (29–32). Disturbances of the function of the DLPFC are associated with depression (33), and moreover, this area has been the target for transcranial magnetic stimulation (TMS) treatment in resistant depression patients (34). This result is also consistent with our preliminary pilot study findings (35).

Another of the significant clusters had a peak in the medial segment of the left superior frontal gyrus (Brodmann area 10), which is part of the ventromedial prefrontal cortex (VMPFC) associated with the regulation of emotions and decision making, including moral judgments (36–39). The role of the VMPFC in mood disorders is also well known (40).

The cluster of activation in the left occipital pole falls within the visual associative cortex (Brodmann area 18), which function is related to the awareness and understanding of visual signals. Since in our case, the stimuli were in the form of a written text, the localization of the activations on the left coincides with other data showing activation of the left associative visual cortex when reading a text (41). Probably, the patients generally retain their attention longer on the DS items than on the DN items.

Limitations of the study might be considered in the small sample size as well as its heterogeneity in terms of nosological diagnosis according to conventional diagnostic standards (bipolar disorder and major depressive disorder). The reported big size cluster (over 700 voxels) in the central cortical region encompasses various distinct functional areas but it is difficult to delineate the specific activations in the relevant subregions. The adopted block design approach in fMRI is more robust experimentally, however, it is relatively distant from clinical reality for application of self-evaluation tools.

In conclusion, we can say that the results confirm the possibility of translational cross-validation of a clinical psychological test (von Zerssen’s depression scale). At this stage, however, we can only confirm the sensitivity of the method (its ability to distinguish healthy controls from depresed patients), but we cannot conclude anything about its specificity (distinction from different psychopathology conditions). For this purpose, it will be necessary in future studies to apply this paradigm to other clinical groups.

## Ethics Statement

This study was carried out in accordance with the recommendations of the Ethical Committee at the Medical University of Plovdiv with written informed consent from all subjects. All subjects gave written informed consent in accordance with the Declaration of Helsinki. The protocol was approved by the “Ethical Committee at the Medical University of Plovdiv.”

## Author Contributions

DS is author of the concept, major parts of the introduction and discussion SK: author of the empirical parts of the study. SB: contributed to the concept, the study design, pilot study and manuscript editing/revisions. R-DS contributed to the methodological framework, selection of the clinical assessment methods. KH contributed to the development of the study design, the pilot study, manuscript editing, and revisions. SK took part in the overall management/supervision of the project.

## Conflict of Interest Statement

The authors declare that the research was conducted in the absence of any commercial or financial relationships that could be construed as a potential conflict of interest. The reviewer SS and handling editor declared their shared affiliation.
